# Background Noise Contributes to Organic Solvent Induced Brain Dysfunction

**DOI:** 10.1155/2016/8742725

**Published:** 2016-01-18

**Authors:** O'neil W. Guthrie, Brian A. Wong, Shawn M. McInturf, James E. Reboulet, Pedro A. Ortiz, David R. Mattie

**Affiliations:** ^1^Cell & Molecular Pathology Laboratory, Communication Sciences and Disorders, Northern Arizona University, Flagstaff, AZ, USA; ^2^Loma Linda VA Medical Center, Loma Linda, CA, USA; ^3^Head & Neck Surgery, Loma Linda University Medical Center, Loma Linda, CA, USA; ^4^Naval Medical Research Unit and Molecular Bioeffects, Wright-Patterson Air Force Base, Dayton, OH, USA

## Abstract

Occupational exposure to complex blends of organic solvents is believed to alter brain functions among workers. However, work environments that contain organic solvents are also polluted with background noise which raises the issue of whether or not the noise contributed to brain alterations. The purpose of the current study was to determine whether or not repeated exposure to low intensity noise with and without exposure to a complex blend of organic solvents would alter brain activity. Female Fischer344 rats served as subjects in these experiments. Asynchronous volume conductance between the midbrain and cortex was evaluated with a slow vertex recording technique. Subtoxic solvent exposure, by itself, had no statistically significant effects. However, background noise significantly suppressed brain activity and this suppression was exacerbated with solvent exposure. Furthermore, combined exposure produced significantly slow neurotransmission. These abnormal neurophysiologic findings occurred in the absence of hearing loss and detectable damage to sensory cells. The observations from the current experiment raise concern for all occupations where workers are repeatedly exposed to background noise or noise combined with organic solvents. Noise levels and solvent concentrations that are currently considered safe may not actually be safe and existing safety regulations have failed to recognize the neurotoxic potential of combined exposures.

## 1. Introduction

Organic solvents are used in a variety of manufacturing industries and they are among the most frequent environmental hazards for factory workers. These solvents include but are not limited to p-xylene, toluene, styrene, ethylbenzene, n-propylbenzene, allylbenzene, *α*-methylstyrene, and trans-*β*-methylstyrene [1, 2]. The main mode of occupational exposure is inhalation, although skin absorption may account for up to 50% of total body burden [3, 4]. The public at large may experience respiratory, skin, and gastric exposures because organic solvents can be found in pharmaceuticals, inks, pesticides, paints, household cleaners, cosmetics, and degreasants [4]. Organic solvents are also constituents of jet propulsion fuel-8 (JP-8) which is the primary fuel consumed by the militaries of the United States and other North Atlantic Treaty Organization countries. Environmental exposure to JP-8 is considered the single most prevalent chemical hazard for military personnel [5]. Other types of jet fuels such as the commercial jet fuels (Jet-A and Jet A-1) that are used by domestic and international airlines are also complex blends of organic solvents. Therefore, commercial aircraft maintenance personnel, aircrew, and even airline passengers are at risk for repeated inhalation exposure [5]. Ultimately, a significant proportion of the world's population is exposed to organic solvents, whether isolated solvents in commercial products or complex mixtures of solvents such as fuels.

It is known that factory workers who are exposed to complex blends of organic solvents may develop cognitive dysfunctions. For instance, impaired attention, memory, and psychomotor functions are among the long-term adverse health outcomes due to solvent exposure [6, 7]. These behavioral findings have helped to promote the hypothesis that exposure to organic solvents (e.g., individual solvents or composite mixtures such as jet fuel) by themselves will alter the function of the central nervous system (CNS). However, this hypothesis is not globally accepted because occupational studies that control for confounding variables such as age and premorbid intelligence could not demonstrate a significant association between a putative exposure and cognitive dysfunction [8, 9]. Furthermore, an analysis of the cumulative weight of the scientific evidence could not support a causal relationship due, in part, to the prevalence of confounding variables in most published studies [10].

A potentially significant confounding variable, which is often overlooked, is the effect of background noise. Workplace environments that contain organic solvents are typically polluted with background noise [11]. For instance, environments within and around factories are contaminated with engine and/or machine noise levels that may range from 70 to 107 decibels (dBA). In some studies focused on solvent induced CNS dysfunctions, care is taken to ensure that workers selected for the studies are not exposed to loud noise that equals or exceeds the legal occupational exposure limit of 90 dB (or the action limit of 85 dBA in the USA and >80 dBA in Europe) referenced to an A-weighted filter (dBA) for an eight-hour work period [12–16]. By limiting the noise exposure to less than eight hours, it is assumed that the noise exhibited little or no effect and this is often verified with audiometric threshold measurements. Indeed, some investigators believe that noise exposure durations that do not permanently elevate audiometric thresholds are safe [17]. In fact repeated exposure to nondamaging noise is currently used as a therapy in certain clinical fields and researchers have been exposing human subjects to noise in order to study the phenomena of noise induced temporary threshold shifts [18–20]. The common belief is that noise is selectively toxic to the preneural sensory cells in the auditory end-organ and if these cells are unaffected by the noise exposure, as evidence by normal thresholds, then the noise is safe to both the ear and the brain. However, some studies have shown that noise exposure could be neurotoxic [21–26]. Therefore, cumulative occupational, residential, and/or recreational air pollution with background noise may act to increase the susceptibility of the CNS to occupational exposure to solvents. As a first approximation to testing this hypothesis, the goal of the current study is to determine whether or not repeated exposure to noise with and without exposure to organic jet fuel would alter brain activity. This goal is further motivated by a previous study on Long-Evans rats, which showed that combined exposure to organic fuel and noise impaired brainstem encoding of stimulus intensity as revealed by the auditory brainstem response [27]. That previous study only assessed brainstem function up to the level of the superior olivary complex. However, the present study employs slow vertex potential recordings that allow for more rostral (midbrain to cortex) assessments.

## 2. Methods

### 2.1. Animals

We have shown previously that male rats are sensitive to the effects of noise and fuel exposures [28, 29]. In the current study, female Fischer344 rats (five weeks old; 55–80 g) were acquired from Charles River Laboratories (Wilmington, MA, USA) and used as subjects. All animals were initially housed in Dayton, Ohio at the Wright Patterson Air Force Base (WPAFB). At the WPAFB, the animals were randomized into four experimental groups (fuel+noise, noise, fuel, and control). There were 10 females per group; however, one animal from the control group was euthanized for pathology screening. The animals were allowed to acclimate to the vivarium for one week. They were then exposed to noise, fuel, or fuel+noise according to their respective grouping. After these exposures, the animals were transferred by air overnight to the Loma Linda Veterans Affairs Medical Center (Medical Center) in California, where they were allowed to recover from their respective exposures for four weeks. This recovery period provided sufficient time for transient effects on the peripheral nervous system to resolve and, thus, not confound central nervous system outcomes [29]. At the end of this four-week convalescent period, the animals received extensive neuroaudiologic assessments and ultimately were euthanized for cytomorphology analyses. The Institutional Animal Care and Use Committees (IACUC) at both the WPAFB and the Medical Center provided oversight and prior approval of all animal protocols.

### 2.2. Jet Fuel Exposure

Organic solvents and solvent blends are particularly toxic to the peripheral auditory system and the presence of such toxicity could affect interpretations related to CNS dysfunctions. Therefore, a particular dosing regimen that has been shown to preserve peripheral function was chosen for the current study [27]. Briefly, the animals received 20 inhalation exposures to 1000 mg/m^3^ of jet propulsion fuel-8 (JP-8). JP-8 is a complex blend of organic solvents and includes the following chemical classes: alkylbenzenes, alkylnaphthalenes, aromatics, and paraffins [28, 29]. The exposure occurred for six hours per day, five days per week for four weeks at the Naval Medical Research Unit-Dayton Inhalation Facility. The Fuels Branch of the US Air Force Research Laboratory provided a single lot of fuel which was used throughout the study. The chemical composition and concentration of the fuel was verified and monitored in real-time throughout the exposure period via Fourier-transform infrared spectrophotometry. These data and further descriptions of the exposure apparatus and protocol have been published previously [27, 30].

### 2.3. Noise Exposure

In the United States, workplace noise exposure is regulated at 90 dBA (with mandatory hearing conservation at 85 dBA) for an eight-hour work period; therefore a somewhat comparable exposure was chosen albeit the exposure time was less than eight hours. Software files were used to generate precisely filtered white noise that were amplified and delivered to the animals in their exposure chambers. The animals were awake and alert in the exposure chambers. They were exposed to a 5.6 to 11.2 kilohertz (kHz) band-pass noise with a symmetric filter-ramp of 48 dB SPL per octave. The noise exposure occurred for six hours per day, five days per week for four weeks. The level of the noise was maintained at 85 dB SPL over the six-hour period and the animals received this noise exposure with or without fuel exposure depending on their respective grouping (e.g., noise-only versus fuel+noise). A Spectral Dynamics Puma data acquisition system (Spectral Dynamics, San Jose, CA) was used to monitor and verify the intensity and spectrum of the noise during the entire exposure epoch.

### 2.4. Neuroaudiology Assessments

#### 2.4.1. Animals

All assessments were conducted inside 6.5′ × 6.5′ or 4′ × 4′ double-walled audiometric booths (Industrial Acoustics Company Inc., Bronx, NY. USA). A cocktail of ketamine/dexdomitor (75/5 mg/kg, i.m.) was used to anesthetize each animal on a 7′′ × 15′′ surgical table with built-in temperature control. Two-channel differential recordings were conducted with a five-electrode array. Subcutaneously implanted electrodes were positioned over the skull (right and left auditory cortex), bilateral mastoids, and in the dorsum close to the tail. Transducer probes with or without microphone assemblies were physically and acoustically coupled to the external auditory meatus of each animal. Acoustic delays introduced by the probe assembly were corrected for each transducer. During neurophysiologic recordings the transducer diaphragm was driven with alternating polarity. The instrumentation used for stimulus presentation, signal acquisition, and manipulation was the Intelligent Hearing System hardware driven by the 3.94b version of the SmartEP Windows USB Software (Intelligent Hearing Systems, Miami, FL).

#### 2.4.2. Auditory Brainstem Response (ABR)

The compound action potential generated as the first fast wave of the ABR was used to obtain neural thresholds to frequency specific stimuli [31–33]. Similar to routine clinical audiology assessments of hearing sensitivity, frequency-specific thresholds were obtained with a modified Hughson-Westlake sequence [27]. Blackman envelopes (1.56 ms) of 512 pure tones were digitally synthesized and presented at a rate of 10/second (sec). Stimulus pure tones were between 2 and 32 kHz in octave (2–4 kHz) and 1/2 octave (6–32 kHz) intervals. The synchronous on-set of neural responses to the pure tones was bandpass filtered between 100 and 3000 Hz and then amplified by 1 × 10^5^. These responses were sampled in sequential 250 microsecond (*μ*s) periods over a 12.5 millisecond (ms) window. Artifact rejection was set at 31 *μ*V and roved 1.3 to 13.1 ms of the recordings. To eliminate any possibility of 60 Hz radiations, each recording was line-filtered.

#### 2.4.3. Slow Vertex Potential (SVP)

The SVP previously referred to as the rat cortical auditory evoked potential was recorded in order to evaluate brain activity. Unlike the rat ABR which measures the first 4.5 ms of brain activity following stimulus onset, the SVP measures brain activity out to 9 ms [34]. Rectangular voltage pulses (clicks) of 100 *μ*s at a rate of 50 Hz were used as stimulus and the average responses from 1024 sweeps were obtained. A sampling rate of 500 *μ*s was employed over a 256 ms recording epoch. The responses were amplified by 1 × 10^5^ and bandpass filtered between 1 and 300 Hz. The intensity dependence of the SVP was plotted by measuring both potential difference (*μ*V: P_2_ minus N_0_) and time of appearance (latency in milliseconds of the P_2_N_0_ complex) as a function of stimulus level. In neuroaudiology assessments, slope indices are known to be reliable biomarkers of CNS pathology [35]. Therefore, slopes of the stimulus response functions were calculated (slope = Δ*Y*/Δ*X*) for each experimental group. The asynchronous postsynaptic origins of the SVP were verified by sequentially increasing the low-pass filter to allow the synchronous presynaptic components of the ABR to vitiate the ascending slope of the SVP [34]. The stimulus dependent-neurogenic origins of both the SVPs and the ABRs were verified by four independent procedures: (1) uncoupling the transducer probe assembly from the pinna, (2) blocking the sound delivery tube, (3) holstering the transducer assembly in a hard-walled coupler, and (4) performing* in situ* recordings on a rat cadaver. These procedures were conducted with the electrodes in place and the animal staged for recording ABRs and SVPs. In all procedures, ABRs and SVPs were absent from the recordings.

The SVP consisted of three positive and one negative component. Neural networks that generate each component can be approximated from the time a given component appears relative to the onset of the stimulus [34]. The positive components were labeled P_0_, P_1_, and P_2_. On average P_0_ occurred at 2 milliseconds (ms) after the onset of the stimulus, which indicates that it is generated from the lower brainstem, at the level of the cochlear nucleus. P_1_ occurred at 3.5 ms which indicates that it represents distributed sources from the superior olivary complex and lateral lemniscus. Therefore, P_0_ and P_1_ are residual fast-waves that vitiate the ascending slope of the SVP which peaks at P_2_ [34]. The P_2_ component exhibited an average latency of 5.5 ms which indicates that it is postsynaptic to the fast waves and generated by the inferior colliculus in the midbrain. The negative component (N_0_) exhibited a long latency of 9 ms which indicates a more central locus of generation. Neurotransmission from the midbrain inferior colliculus to the cortex is ~3.1 ms in the rat which approximates the signal conduction time (3.5 ms) between P_2_ and N_0_ [34]. This P_2_N_0_ potential is considered the major component of the rat long latency auditory evoked potential [36].

#### 2.4.4. Distortion Product Otoacoustic Emissions (Emissions)

Emissions assess the function of preneural sensory cells, called cochlear outer hair cells. Anesthetized (ketamine/dexdomitor 75/5 mg/kg, i.m.) animals were placed on a heated surgical table and their body temperature maintained at 37°C. An emissions probe assembly consisting of radial horn tweeters (Radio Shack, Tandy Corp, Fort Worth, TX) and an ER-10B+ microphone (Etymotic Research, Elk Grove Village, IL) was extended via tubing and fitted to the external auditory meatus via an ER3-34 infant silicon tip (Etymotic Research). Stimulus presentation, response acquisition, and analysis were controlled with a customized algorithm written in LabVIEW version 7.1 (National Instruments, Austin, TX). Emissions magnitude as a function of increasing stimulus levels (*L*_2_) were measured for stimulus frequencies (*f*_2_) of 4, 6, 8, 11, 16, 24, and 32 kHz. Frequency-specific emission thresholds were determined from these measurements as the lowest *L*_2_ level that elicited an emission that was greater than two standard deviations above the mean noise floor. Emission thresholds are often used in both humans and animals to assess the sensitivity of the sensory cells [37, 38]. Furthermore, in humans, emission thresholds have been shown to correlate with behavioral audiometric thresholds [38, 39]. The *f*_2_/*f*_1_ ratio was 1.25 and *L*_2_ = *L*_1_ − 10. During each measurement *L*_2_ increased in 5 dB steps from 10 to 75 dB sound pressure level (SPL). Emissions measurements were calibrated in a 0.2 cm^2^ hard-walled cavity that approximates the rat's external auditory meatus volume.

### 2.5. Cytomorphology

The cytomorphology work was identical to that described in a previous study [27]. Briefly, anesthetized animals were euthanized at the end of the neuroaudiology procedures and their cochleae were fixed* in situ* by perfusing 4% formaldehyde through the perilymphatic scala. Each cochlea was then removed and further fixed overnight in the same fixative. The cochlear neurosensory epithelium was micro-dissected, cleared in glycerol, and mounted on microscope slides. To detect damaged/missing cells, differential interference contrast microscopy was employed to examine each 0.33 millimeter segment of the neurosensory epithelium [27, 40–42]. The number of outer hair cells along the spiral longitudinal axis of the epithelium was counted. These cell counts were used to construct cytocochleograms that plot the percentage of cells present as a function of percentage distance along the length of the neurosensory epithelium. Outer hair cells were quantified because it is known that organic solvents are particularly toxic to these cells [30, 43, 44].

### 2.6. Statistical Calculations

Statistical calculations were performed with Prism 5, version 5.03 (GraphPad Software, Inc., La Jolla, CA, USA). The null hypothesis was that there is no difference in brain responsiveness between the control and exposure groups. The exposure groups consisted of animals that were exposed to noise-only, fuel-only, or fuel+noise. The control group was placed in the exposure chamber on a daily basis (similar to the exposure groups) but was not exposed to noise or fuel. All data sets except for the slope data were treated with a mixed-model analysis of variance (ANOVA) design. Separate two-way ANOVAs were computed for the emission and ABR threshold data. Grouping (control, noise, fuel, and fuel+noise) served as the between-group factor and frequency (2 to 32 kHz) served as the within-group factor. Two-way ANOVAs were also calculated for the response times and response potential data, such that grouping (control, noise, fuel and fuel+noise) served as the between-group factor and stimulus level (100 to 40 dB SPL) served as the within-group factor. To determine pairwise differences between groups, separate group (control versus an exposure group) by stimulus level ANOVAs was computed for the response time data and the response potential data. These pairwise ANOVA computations are presented in the figure panels. *F*-tests were computed to determine significant differences between the slopes of the control group compared to that of an experimental group and these pairwise data are also presented in the figure panels. A *P*-value of <0.05 was defined as statistically significant.

## 3. Results

### 3.1. Normal Thresholds

**Figure 1:**
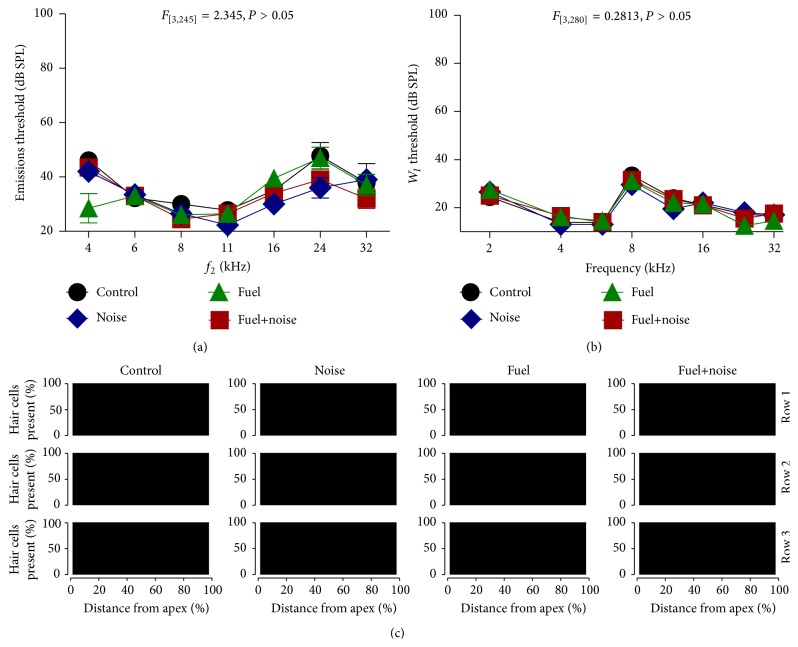
Normal thresholds. (a) Emissions thresholds revealed no major differences in preneural sensitivity between the experimental groups. (b) Synchronous compound action potential (*W*_*I*_) also revealed no major differences in neural sensitivity between the experimental groups. (c) Cytocochleograms showed no loss (all black) of sensory cells (outer hair cells) as a function of distance along the neurosensory epithelium. Error bars in this and all figures are ±1 standard error.

The threshold for preneural sensory cells was determined for each experimental group. Stimulus induced transduction from these cells generates acoustic emissions that can be measured at subthreshold to super-threshold levels of stimulation for discrete stimulus frequencies.  Figure 1(a) reveals that there were no major differences in emissions thresholds between the nonexposed (control) and the exposure groups. This suggests that outer hair cell sensitivity was preserved. A two-way ANOVA calculation revealed that there were no significant differences (*F*_3,245_ = 2.35, *P* = 0.07) in emission thresholds between the groups. The synchronous compound action potential generated from the cochlear nerve and revealed as the first fast-wave (*W*_*I*_) of the ABR was measured for each experimental group. The threshold of this neural response was then plotted as a function of stimulus frequency.  Figure 1(b) demonstrates that there were no major differences in threshold between the nonexposed and exposure groups. A two-way ANOVA calculation revealed that there was no significant differences (*F*_3,280_ = 0.28, *P* = 0.84) in ABR *W*_*I*_ thresholds between the groups. Therefore, both the preneural (emissions) and neural (ABR) data suggest that neither the noise or fuel exposures resulted in a detectable change in end-organ sensitivity. These functional data were further supported by cytomorphology studies of the end-organ. For instance, Figure 1(c) shows cytocochleograms that plot the proportion of sensory cells as a function of distance along the neurosensory epithelium. Note that no damaged or missing/dead cells were detected. Therefore, the combined results indicate that the fuel and noise exposures failed to induce detectable lesions in the peripheral auditory nervous system.

### 3.2. Abnormal Slow Vertex Potential (SVP)

**Figure 2:**
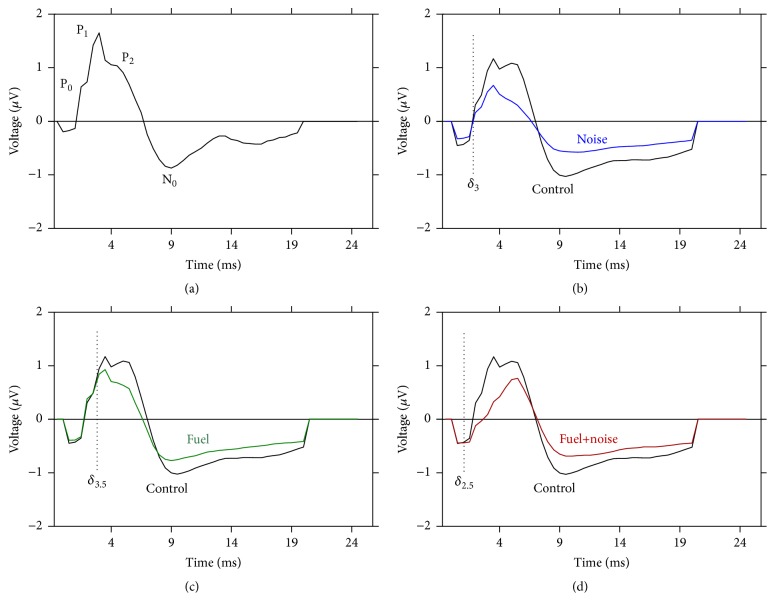
Abnormal SVP. (a) A typical SVP with three positive components (P_0_, P_1_, and P_2_) followed by a negative component (N_0_). (b) Grand average SVP from the control group compared with that from the noise exposed group. Note that the noise exposure suppressed the amplitude of P_1_, P_2_, and N_0_ which resulted in a truncated waveform. (c) Similar, but less severe outcomes were observed after fuel-only exposure. (d) Combined exposure to both the fuel and noise resulted in a loss of P_0_ and P_1_ combined with suppression of P_2_ and N_0_. Furthermore, the overall waveform is truncated. The vertical dotted lines demark the point in time where the instantaneous voltage trace deviated (*δ*) from normal. Note that fuel+noise exposure exhibited the earliest deviation at 2.5 ms. These instantaneous voltage traces were recorded with a 70 dB SPL rectangular voltage pulse.

Figure 2(a) shows a representative SVP from a randomly selected recording from the control group. This recording illustrates the various positive and negative components as well as the overall morphology of the waveform.  Figure 2(b) compares the grand average recordings from the control and noise exposed groups. After noise exposure, there seems to be significant distortions in the morphology of the waveform. A similar effect was observed following fuel-only exposure (Figure 2(c)), although the effects were less severe than that observed after noise-only exposure. The most severe waveform distortions occurred following exposure to both the fuel and noise (Figure 2(d)). These waveform distortions suggest that the noise exposure may alter the responsiveness of the brain and fuel exposure by itself may mimic this effect at a more modest level of severity. Furthermore, there seems to be a synergistic negative effect when the fuel and noise are combined.

**Figure 3:**
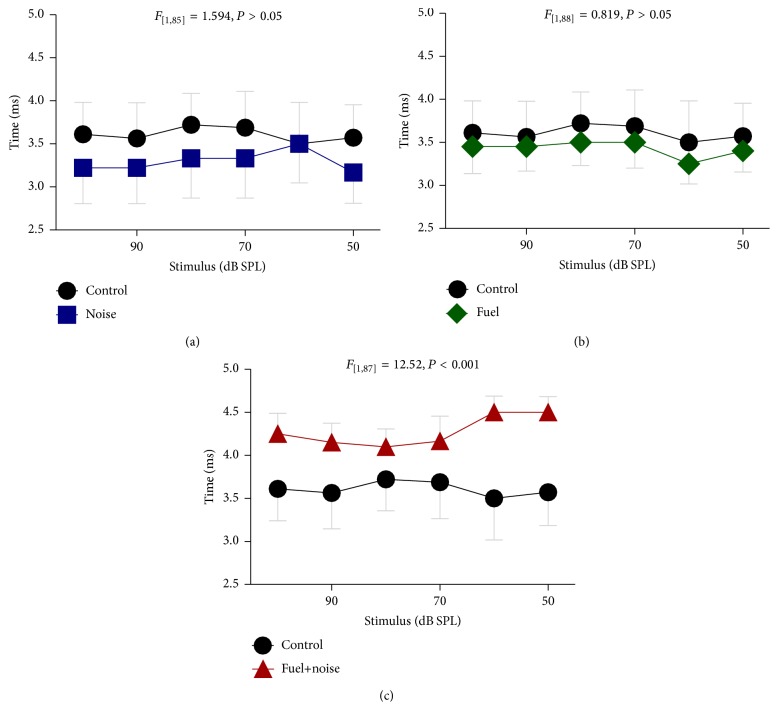
Abnormal conduction time. The time difference between P_2_ and N_0_ was measured for each exposure group and compared to that of the nonexposed (control) group. Both the (a) noise and (b) fuel exposed groups exhibited conduction times that were generally similar to that of the control group. However, the fuel+noise group exhibited major delays in conduction time (c).

To quantify these observations, the response time and magnitude for the P_2_N_0_ complex were measured. The P_2_N_0_ complex was the most robust of all the components and could be recorded with stimulus levels as low as 40 dB SPL. Therefore, stimulus response growth functions could be plotted for each animal within the experimental groups.  Figure 3 plots the latency of the P_2_N_0_ complex as a function of stimulus level. Mild differences between the control group and the noise-only or the fuel-only groups could be detected. However, there was a major difference between the control and fuel+noise groups. This suggests that combined exposure to fuel+noise may alter neural conduction time. A two-way ANOVA revealed that there was a significant difference (*F*_3,178_ = 9.33, *P* = 0.001) in neural conduction time between the groups but a statistically significant difference was only evident between the control and fuel+noise groups (*F*_1,87_ = 12.52, *P* = 0.001). Taken together, the results imply that combined exposure to fuel+noise may reduce neural conduction time.

**Figure 4:**
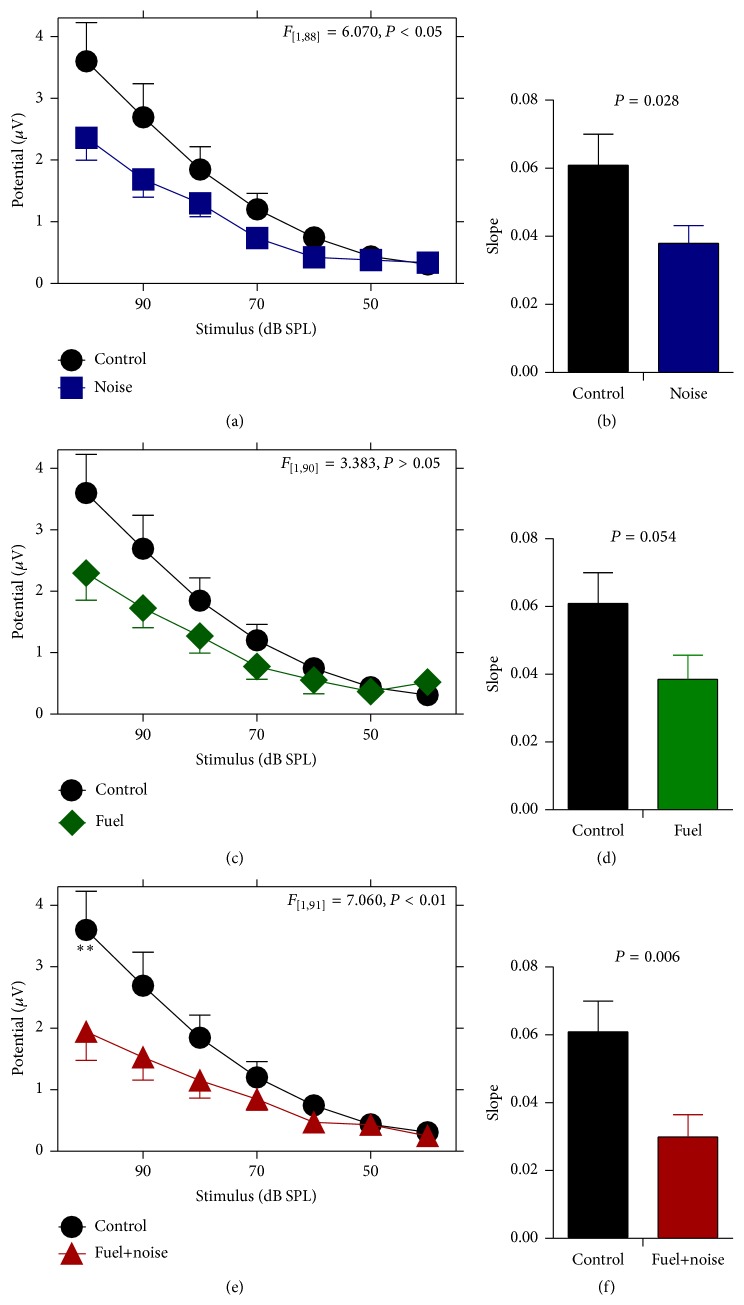
Abnormal response magnitude. The potential difference between P_2_ and N_0_ was measured for each exposure group and compared to that of the nonexposed (control) group. (a) After noise exposure there was a reduction in stimulus response magnitude across a wide range of stimulus levels. (b) This was further confirmed with a truncated stimulus response growth rate (slope). (c and d) Similar, but less severe outcomes were observed after fuel-only exposure. (e and f) Combined exposure to fuel and noise resulted in a significant reduction in stimulus response magnitude which was further supported by a truncated slope.

The magnitude of the P_2_N_0_ complex was also measured to further characterize the brain's responsiveness as a result of the exposures.  Figure 4 demonstrates that the noise exposure reduced the magnitude of the P_2_N_0_ complex. For instance, the magnitude of the complex is depressed over a wide range of stimulus levels, particularly for high levels of stimulation. Furthermore, the slope of the stimulus response growth function for the noise group was compressed relative to that of the control group. This indicates that the noise exposure altered neural activity. Exposure to the fuel also reduced the magnitude of the P_2_N_0_ complex and compressed the slope of the stimulus response growth function but these effects were not as severe as the effect of the noise exposure. Combined exposure to the fuel and noise resulted in magnitude reductions of the P_2_N_0_ complex along with a truncated stimulus response slope. Therefore, noise exposure by itself or fuel+noise exposure may alter neural responses. A two-way ANOVA calculation revealed that there was a significant difference (*F*_3,185_ = 3.36, *P* = 0.02) in response magnitude between the groups. Pairwise comparisons further revealed there was no significant difference between the control and fuel groups (*F*_1,90_ = 3.38, *P* = 0.07), but there were significant differences between the control and noise (*F*_1,88_ = 6.07, *P* = 0.02) and control and fuel+noise (*F*_1,91_ = 7.06, *P* = 0.01) groups. These results further suggest that the noise and fuel+noise exposures may alter neural responses.

## 4. Discussion

It is believed that repeated occupational exposure to organic solvents may lead to CNS dysfunctions [12–16]. This line of thinking is reasonable given that solvents have been shown to be neurotoxic in animal experiments [45]. However, organic solvent induced CNS dysfunctions have been contested and several studies could not demonstrate a causal relationship [8–10]. In the current study, exposure to JP-8 which is a complex blend of organic solvents resulted in no statistically (*P* > 0.05) significant changes in asynchronous neurotransmission from the midbrain to the cortex as revealed by the SVP. A previous JP-8 exposure study evaluated synchronous neurotransmission within the lower brainstem (cochlear nucleus and superior olivary complex) of Long-Evans rats and found a significant effect [27]. Another study on Fischer344 rats found that synchronous neurotransmission was impaired in the superior olivary complex but not the cochlear nucleus [28]. Therefore, specific types of neural networks might be more functionally vulnerable than others which might help to explain conflicting results between studies. Important to the current study is whether neural networks that are less vulnerable to JP-8 become more vulnerable after exposure to background noise.

The current study evaluated whether or not repeated exposure to 85 dB SPL of noise for six hours alone or combined with organic fuel could affect the neurophysiology of the brain. The main findings indicate that the noise exposure, by itself, resulted in an inhibition of brain responsiveness as demonstrated by reductions in the SVP magnitude as stimulus level increased. This effect was exacerbated with inhalation exposure to the fuel. Furthermore, combined exposure to the noise and fuel produced a significant signal transmission deficit as revealed by increased latency of the SVP across a wide range of stimulus intensities. These abnormal neurophysiologic findings occurred in the absence of hearing loss and detectable damage to auditory sensory cells. Taken together, the results suggest that the functions of neural networks that show little or no vulnerability to organic jet fuel exposure may eventually become vulnerable after combined exposure to fuel and background noise.

Human exposure to background noise is ubiquitous in occupational environments that are polluted with organic solvents, yet the neurotoxic capacity of noise by itself or in combination with solvents has not received much attention. The National Institute for Occupational Safety and Health and the Occupational Safety and Health Administration limits workplace noise exposure to less than 85 dBA and 90 dBA over an 8-hour work period because such exposure limits do not promote permanent hearing loss among most workers. The observations from the current work have significant implications for all occupations where workers are repeatedly exposed to legally safe levels of noise or safe noise levels combined with legally safe concentrations of organic solvents. This is because what is currently considered safe may not actually be safe. Current government and industry regulations have failed to establish guidelines regarding the interaction between background noise and permissible levels of solvents. Furthermore, current occupational hearing conservation programs do not consider the neurotoxic potential of daily exposure to workplace noise. In the current study, noise-only exposure resulted in a significant reduction in brain responsiveness. Beyond implications for occupational settings, this particular finding is significant to several areas of human health and well-being. For instance, ear-level noise masking and sound therapy are used in long-term treatment of patients who have normal hearing but suffer with tinnitus (constant ringing in the ears or head) and/or hyperacusis (abnormal sound tolerance) [46, 47]. However, no attention is given to whether a side effect from such noise therapy is altered brain function that could have unintended consequences. Furthermore, it is estimated that 80–90% of children listen to personal listening devices for a significant proportion of their day, but unlike hearing loss, little attention is given to the neurotoxic effects from such sound exposures [48].

Experiments conducted on workplace noise exposure are usually focused on pathological consequences following loud/damaging noise. Relatively little attention is focused on pathological consequences following exposure to moderate/nondamaging noise. However, a recent series of experiments conducted on adult cats have shown that weekly exposure to nondamaging noise can decrease the responsiveness of neurons in the thalamus and the primary auditory cortex as revealed by local field potentials [22–24]. The neural suppression occurred among neurons that were selective for frequencies within the bandwidth of the noise, an indication that the loss of neural responsiveness is specific to the noise exposure. This loss is permanent and is considered to reflect abnormal reorganization of thalamocortical neural networks. Noise induced suppression of neural activity occurred with narrow (2–4 kHz), wide (4–20 kHz) and third-octave bands of noise. Furthermore, the suppression could be induced with noise levels as low as 68 dB SPL. These previous results from the cat support the current results on the rat, where noise exposure impaired stimulus-intensity gating of asynchronous neural networks as revealed by the SVP. The significance of these observations can be inferred from similar observation on humans. For instance, noise induced suppression of brain activity has been shown to be associated with cognitive deficits in memory, attention, and psychomotor tasks as well as cortical hemispheric reorganization [21, 25]. Further work that isolates neural pathway function deficits affecting behavior are needed to determine if other sensitive CNS regions are degraded with combined solvent and noise exposures. Ultimately, these CNS alterations may underlie the known association between workplace noise exposure and cognitive performance decrements including task related error rates and increased rates of accidents [49].

## 5. Conclusion

The current study revealed that repeated exposure to noise can alter responsiveness of the brain. This result may have implications for residential, recreational, and occupational environments that are polluted with background noise (e.g., day and night time neighborhood noises; road noise during a commute to and from work; and machine/engine noises at work). The current study also suggests that noise and organic fuel exposures may interact to alter brain activity. This might be important to occupational observations that infer a relationship between organic solvent exposure and cognitive deficits. This correlative observation is important because noise pollution is ubiquitous and the amount of nonoccupational noise exposure combined with workplace noise exposure may exacerbate putative associations between solvent intoxication and cognitive deficits. Given that the current study focused on an animal model of brain dysfunction, future studies on human participants are now needed to further clarify the relevance of such dysfunction. For instance, it would be relevant to know what cognitive (behavioral) outcomes can be expected from individuals with brain dysfunctions that are similar to what was observed in the current study.
